# Impact of Preoperative Sarcopenia on Patient Survival After Esophagectomy for Cancer: A Retrospective Cohort Study

**DOI:** 10.1245/s10434-025-18620-y

**Published:** 2025-10-25

**Authors:** Alberto Aiolfi, Davide Bona, Gianluca Bonitta, Quan Wang, Domenico Albano, Alberto Luporini, Giuseppe Banfi, Luca Maria Sconfienza, Luigi Bonavina

**Affiliations:** 1 I.R.C.C.S. Ospedale Galeazzi – Sant’Ambrogio, Division of General Surgery, Department of Biomedical Science for Health, University of Milan, Milan, Italy; 2 Department of Biomedical Sciences for Health, Division of General and Foregut Surgery, University of Milan, Milan, Italy

**Keywords:** Esophageal adenocarcinoma, Sarcopenia, Psoas Muscle Index, Survival

## Abstract

**Introduction:**

The prevalence of sarcopenia exhibits considerable variation depending on patient age, definitions, diagnostic techniques, and classifications. Previous studies showed that sarcopenia in patients with esophageal cancer might increase the risk of postoperative complications. However, its impact on overall (OS) and disease-free survival (DFS) is unclear.

**Methods:**

Retrospective multicenter study (January 2014 to December 2024), including patients with resectable Siewert I-II esophageal adenocarcinoma who underwent Ivor-Lewis esophagectomy. Sarcopenia was assessed by using the Psoas Muscle Index (PMI), calculated at the level of the third lumbar vertebra on preoperative CT scan.

**Results:**

Overall, 338 patients were included; 79.5% were male, and the median age was 66 years. The prevalence of sarcopenia was 39.7%. Anastomotic leak (21.6% vs. 10.8%; *p* = 0.02), pneumonia (14.9% vs. 6.4%; *p* = 0.02), and 90-day mortality (7.5% vs. 1.9%; *p* = 0.03) were significantly higher in sarcopenic patients. On multivariate analysis, sarcopenia was an independent predictor of anastomotic leak (odds ratio [OR] 1.41, 95% confidence interval [CI] 1.12–1.87), pneumonia (OR 1.84, 95% CI 1.24–2.15), and 90-day mortality (OR 1.21, 95% CI 1.05–1.55). The 60-month DFS (32% vs. 52%; *p* = 0.001) and OS (47% vs. 61%; *p* = 0.008) were significantly reduced in sarcopenic patients. Sarcopenia was an independent predictor of poor survival in the regression analysis (hazard ratio [HR] 1.84, 95% CI 1.36–2.78).

**Conclusions:**

Sarcopenia is a highly prevalent condition among patients with esophageal adenocarcinoma. Patients with sarcopenia have lower DFS and OS rates compared with those without sarcopenia. Sarcopenia was independently associated with postoperative anastomotic leak, pneumonia, 90-day mortality, and poor long-term survival.

Esophageal cancer ranks as the seventh most prevalent malignancy globally and stands as the sixth leading cause of cancer-related mortality.^1^ Despite significant advancements in multimodal treatments and the growing evidence indicating that older patients may safely undergo esophagectomy, overall prognosis remains poor. The reported 5-year net survival rates vary between 14 and 23.5%.^2,3^ This highlights the critical need for identifying modifiable preoperative risk factors that could be targeted before esophagectomy to enhance patient outcomes.

Sarcopenia, characterized by loss of skeletal muscle mass and strength, is often associated with aging, frailty, and increased morbidity in the elderly population.^4,5^ Sarcopenia occurs independently of body mass index (BMI); thus, individuals may present with a normal BMI or even obesity, which can obscure the underlying loss of muscle mass. This is the reason why body impedance analysis, assessment of muscle strength and gait speed, and assessment of muscle mass by ultrasound or computed tomography scan are necessary to correctly identify individuals with sarcopenia.^5,6^ Notably, patients with objectively assessed sarcopenia have demonstrated worse long-term survival rates in various cancer types, including pancreatic, colorectal, and liver cancer.^7–9^ In the context of multimodal treatment of esophageal cancer, sarcopenia is often associated with dysphagia and malnutrition and has more recently emerged as a modifiable preoperative risk factor.^10^ A recent meta-analysis highlighted that sarcopenia as a significant predictor of inferior overall survival (OS) and disease-free survival (DFS) after esophagectomy.^11^

In this study, we hypothesized that the presence of sarcopenia can adversely impact the outcomes of esophagectomy for cancer. We aimed to assess the correlation between preoperative sarcopenia, evaluated through computed tomography (CT), and short- and long-term outcomes of esophagectomy.

## Materials and Methods

This study involved a retrospective analysis conducted across two specialized esophageal cancer centers in Italy (IRCCS Policlinico San Donato and IRCCS Ospedale Galeazzi-Sant’Ambrogio). Consecutive patients who underwent esophageal resection for esophageal adenocarcinoma between January 2014 and December 2024 were included in the analysis. The study was performed in accordance with the Declaration of Helsinki and after approval of the local institutional review board (IRB). Specific consent was waived, and the data were anonymized.

### Eligibility Criteria

The study included all patients diagnosed with Siewert type I or II esophageal adenocarcinoma who underwent R0 hybrid or total minimally invasive Ivor-Lewis esophagectomy, with or without prior neoadjuvant therapy according to the CROSS or FLOT protocols, with immunotherapy added in select cases. Esophageal resection was scheduled 6 to 8 weeks after the completion of neoadjuvant therapy and CT restaging as per current ESMO guidelines.^12^ Criteria for exclusion were the following: high-grade dysplasia in Barrett’s esophagus, Siewert type III tumors, squamous cell esophageal carcinoma, patients with distant metastases or concomitant malignancies, or comorbidities that could potentially influence body composition. All surgical interventions were conducted by two proficient surgeons; each having performed over more than100 esophagectomies before the study period.

### Study Population and Baseline Assessment

The following data were retrieved: age, gender, body mass index (BMI), American Society of Anesthesiologists (ASA) score, Charlson comorbidity index (CCI), tumor characteristics (e.g., histology, TNM classification, pathological stage), tumor location, surgical procedure and approach (e.g., hybrid or MIE), use of neoadjuvant and/or adjuvant therapy, duration of follow-up, postoperative outcomes and complications, and long-term survival.

### Outcomes Measurement and Definition of Sarcopenia

The primary outcomes were survival benefit, including OS and DFS. Overall survival was defined from esophagectomy until death from any cause. The recurrence of local esophagus and locoregional lymph nodes and the metastasis of distant lymph nodes and distant organs were identified as disease-related progression. Disease-free survival was calculated from the day of surgery to the date of disease-related progression or death. Staging was performed according to the American Joint Committee on Cancer/Union for International Cancer Control (AJCC/UICC) staging system (8th edition).^13^ Secondary outcomes were postoperative complications defined in accordance with the Esophagectomy Complications Consensus Group guidelines.^14^ Sarcopenia was assessed by using staging CT during the preoperative period in patients who did not receive neoadjuvant therapy. For patients who received neoadjuvant therapies, restaging CT scans after completion of preoperative treatment were utilized for evaluation. A patient undergoing neoadjuvant treatments was classified as sarcopenic if the PMI was below the threshold on post-neoadjuvant therapy imaging. CT scans were reviewed by two expert radiologists to evaluate the total cross-sectional transverse areas of the psoas muscle at the level of the third lumbar vertebra (L3). The psoas muscle index (PMI) was calculated at the level of the lumbar vertebra, normalized by height, and reported in cm^2^/m^2^. Sarcopenia was defined using sex-specific cutoff values, with thresholds for PMI set at 5.3 cm^2^/m^2^ for men and 3.6 cm^2^/m^2^ for women.^15^

### Surgical Technique

Hybrid Ivor-Lewis (IL) esophagectomy was performed with a five-port laparoscopic approach combined with a posterolateral right thoracotomy through the fifth intercostal space. During the laparoscopic phase, the right gastroepiploic arcade was entirely preserved and a 4-cm–wide gastric conduit was fashioned using a mechanical stapler. A D2-celiac lymphadenectomy was systematically performed. During the thoracotomy phase, the esophagus was mobilized en bloc with the periesophageal fatty tissue from the level of azygos arch to the diaphragm, and an infracarinal lymphadenectomy was performed. The gastric conduit was gently maneuvered into the right hemithorax, and an esophagogastric end-to-side anastomosis was performed at the level of the azygos vein by using a 25 mm circular stapler. For the total minimally invasive esophagectomy, a right thoracoscopy as performed in a semiprone or prone position.^16^

### Statistical Analysis

Categorical variables were presented as absolute and percentage frequencies, whereas continuous variables were expressed as median values along with interquartile ranges (IQR). The Wilcoxon rank-sum test or the chi-square test were performed as appropriate. The Kaplan-Meier analysis and the Cox regression were performed to estimate OS and DSF parameters. The Cox regression assumptions were checked by dedicated diagnostics, in particular the proportional hazard assumption was inspected by using Schoenfeld residuals. Diagnostic evaluation of the Cox regression indicated no violations of the pre-analysis assumptions. The logistic regression was also performed for anastomotic leak (AL), pneumonia, and 90-day mortality. All confidence intervals were computed at 95%, and the statistical significance was defined when two-sided *p* value ≤ 0.05. The statistical analysis was conducted by using R software version 4.5.1 from the R Foundation in Vienna, Austria.^17^

## Results

During the study period, 385 patients underwent IL esophagectomy for Siewert type I-II esophageal adenocarcinoma. Forty-seven patients were excluded from analysis, because no presurgical CT scan was available; 338 patients were included in the final analysis. Overall, 79.5% of patients were males (median age 66 [range 32-89] years) (Table 1). Based on CT scan parameters, 134 patients (39.7%) exhibited sarcopenia. The median age was greater in the sarcopenic group (69 vs. 63.6 years, *p *< 0.001), and the median BMI was lower in the sarcopenic group. The rates of neoadjuvant chemoradiotherapy and hybrid IL esophagectomy did not differ between groups. Tumor stage was reported according to the AJCC 8th edition was equally distributed in the two groups (Table 1). The median number of harvested lymph nodes and positive lymph nodes were comparable in the two groups.

**Table 1:** Demographics and histopathologic data of the patient population

	Sarcopenic (n = 134)	Nonsarcopenic (n = 204)	*p*
Age (yr)	69 (34-89)	63.6 (32-79)	<0.001
Gender, male	108 (80.6%)	160 (79%)	0.73
BMI (kg/m^2^)	23.9 (17.4-37.2)	25.6 (17.9-41.1)	0.015
**Comorbidities**			
Hypertension	55 (41%)	89 (44%)	0.83
Arrhythmia/atrial fibrillation	13 (9.7%)	14 (6.9%)	0.41
CAD	21 (15.7%)	29 (14.3%)	0.76
Diabetes	20 (14.9%)	35 (17.2%)	0.77
Smoking	18 (13.4%)	30 (14.8%)	0.87
COPD	20 (15%)	27 (13.3%)	0.75
CVA	4 (2.9%)	3 (1.5%)	0.44
TEE	5 (3.7%)	3 (1.5%)	0.27
**Neoadjuvant therapy**	83 (61.9%)	124 (61.1%)	0.92
Immunotherapy	27 (20.1%)	43 (21.1%)	0.89
**Surgery type**			0.72
Hybrid	105 (78.3%)	152 (74.8%)	
MIE	29 (21.7%)	51 (25.2%)	
**Tumor location**			0.53
Siewert I	56 (41.7%)	82 (40.4%)	
Siewert II	78 (58.3%)	121 (59.6%)	
**Tumor stage (p or yp)**			
0	6 (4.5%)	8 (3.9%)	0.54
I	20 (14.9%)	30 (14.8%)	0.86
II	17 (12.7%)	28 (13.8%)	0.39
III	59 (44%)	85 (41.8%)	0.29
IV	32 (23.9%)	52 (25.6%)	0.51
**Tumor grade**			
Well differentiated (G1)	9 (6.7%)	15 (7.4%)	0.51
Moderately differentiated (G2)	80 (59.7%)	126 (62.1%)	0.49
Poorly differentiated (G3)	45 (33.6%)	62 (30.5%)	0.29
Nodes harvested	22 (5-62)	24 (6-58)	0.31
Nodes positive	1 (0-15)	1 (0-16)	0.29
LOS (days)	9 (6-19)	11 (8-43)	0.26
OT (min)	290 (254-481)	316 (264-502)	0.19

Data are presented as media (interquartile range IQR) or number (percentages) as appropriate

*BMI* body mass index; *CAD* coronary artery disease; *COPD* chronic obstructive pulmonary disease; *CVA* cerebrovascular accident; *TEE* thromboembolic event; *MIE* minimally invasive esophagectomy

In the univariate analysis, patients with sarcopenia had a higher rate of AL (21.6% vs. 10.8%; *p* = 0.02), pneumonia (14.9% vs. 6.4%; *p* = 0.02), overall complications (52.2% vs. 38.8%; *p* = 0.01), and 90-day mortality (7.5% vs. 1.9%; *p* = 0.03) (Table 2). On multivariate analysis, sarcopenia was an independent predictor of AL (OR 1.41, 95% CI 1.12–1.87) (Table 3), pneumonia (OR 1.84, 95% CI 1.24–2.15) (Table 4), and 90-day mortality (OR 1.21, 95% CI 1.05–1.55) (Table 5).

**Table 2:** Postoperative complications

	n = 338	Sarcopenic (n = 134)	Nonsarcopenic (n = 204)	*p*
Anastomotic leak	51 (15.1%)	29 (21.6%)	22 (10.8%)	0.02
Pleural effusion (R/L)	91 (27%)	35 (26.1%)	56 (27.6%)	0.88
Pneumothorax	34 (10.2)	14 (10.4%)	20 (9.8%)	0.97
Pneumonia	33 (9.8%)	20 (14.9%)	13 (6.4%)	0.02
ARDS	8 (2.4%)	3 (2.2%)	5 (2.5%)	0.94
Chylothorax	4 (1.2%)	2 (1.5%)	2 (0.9%)	0.98
Cardiac arrythmia	40 (11.8%)	17 (12.7%)	23 (11.3%)	0.82
DVT/PE	6 (1.8%)	2 (1.5%)	4 (1.9%)	0.89
Deep/superficial SSI	15 (4.4%)	5 (3.7%)	10 (4.9%)	0.81
Sepsis/MOF	10 (2.9%)	5 (3.7%)	5 (2.5%)	0.72
Overall complications	148 (43.9%)	70 (52.2%)	78 (38.3%)	0.01
90-day mortality	14 (4.1%)	10 (7.5%)	4 (1.9%)	0.03

*R* right; *L* left; *ARDS* acute respiratory distress syndrome; *DVT* deep vein thrombosis; *PE* pulmonary embolism; *SSI* surgical site infection; *MOF* multiorgan failure

Data are presented as number (percentage)

**Table 3:** Logistic regression analysis for anastomotic leak

	OR	95% CI
Age (yr)	1.02	0.91-1.11
P stage^a^		
0-I	Ref	Ref
II	0.69	0.24-1.61
III	1.05	0.55-1.94
IV	0.98	0.68-1.74
Surgical approach (MIE)	1.05	0.75-1.36
Neoadjuvant CR therapy	1.26	0.86-1.45
Sarcopenia	1.41	1.12-1.87

^a^ pTNM and ypTNM

*OR* odds ratio; *CI* confidence interval

**Table 4:** Logistic regression analysis for pneumonia

	OR	95% CI
Age (yr)	1.09	0.85-1.19
P stage^a^		
0-I	Ref	Ref
II	0.94	0.65-1.69
III	1.16	0.85-1.74
IV	1.41	0.89-2.03
Surgical approach (MIE)	0.75	0.435-1.06
Neoadjuvant CR therapy	1.15	0.75-1.37
Sarcopenia	1.84	1.24-2.15

^a^ pTNM and ypTNM

*OR* odds ratio; *CI* onfidence intervals

**Table 5:** Logistic regression analysis for 90-day mortality

	OR	95% CI
Age (yr)	1.12	0.78.1.26
P stage^a^		
0-I	Ref	Ref
II	1.14	0.46-1.81
III	1.26	0.89-1.81
IV	1.22	0.74-1.56
Surgical approach (MIE)	0.87	0.61-1.31
Neoadjuvant CR therapy	1.19	0.87-1.25
Sarcopenia	1.21	1.05-1.55

^a^ pTNM and ypTNM

*OR* odds ratio; *CI* confidence interval

**Fig. 1:**
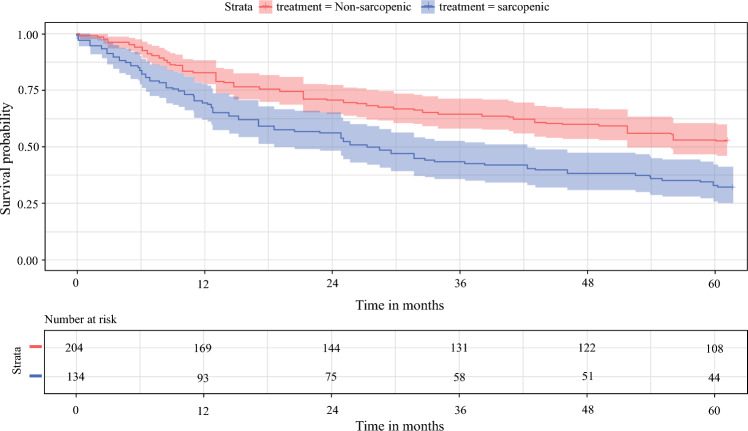
Kaplan-Meier survival curve for DFS for sarcopenic (blue line) vs. nonsarcopenic (red line) patients. The continuous line represents the median, while the green and red shadow represent confidence intervals. The X-axis represent postoperative follow-up time (month). The Y-axis represent the survival probability. The number of patients at risk is indicated below

**Fig. 2:**
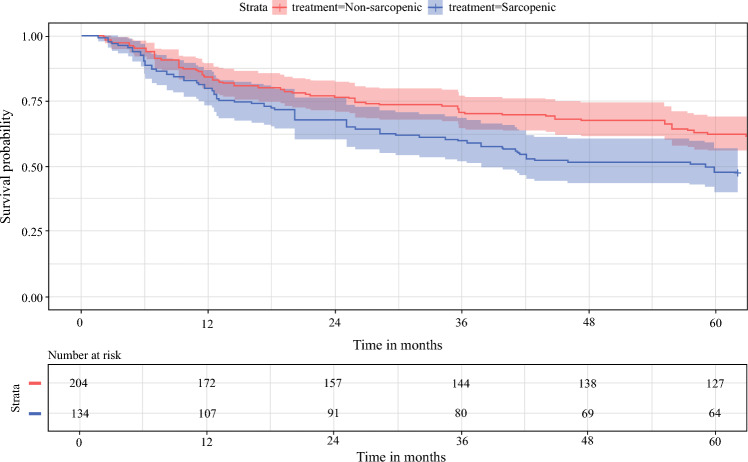
Kaplan-Meier survival curve for OS for sarcopenic (blue line) vs. nonsarcopenic (red line) patients. The continuous line represents the median, while the green and red shadow represent confidence intervals. The X-axis represent postoperative follow-up time (month). The Y-axis represent the survival probability. The number of patients at risk is indicated below

The 60-month DFS was significantly lower in the sarcopenia (32.1%, 95% CI 25.1–41.2) vs. no sarcopenia group (52.5%; 95% CI 46.1-59.8) (*p* = 0.001) (Fig. 1). Similarly, the 60-month OS was significantly lower in the sarcopenia (47%, 95% CI 41.2-57.6) vs. no sarcopenia group (61%; 95% CI 55.3-69.1) (*p* = 0.008) (Fig. 2). Sarcopenia (HR 1.84, 95% CI 1.36–2.78), AL (HR 1.46, 95% CI 1.15–2.94), pneumonia (HR 2.26, 95% CI 1.41–3.05), stage IV disease (HR 1.95, 95% CI 1.45–2.85) were independent predictor of poor survival in the logistic regression analysis (Table 6).

**Table 6:** Cox regression analysis for OS

	HR	95% CI
Age (yr)	1.08	0.75-1.28
P stage^a^		
0-I	Ref	Ref
II	0.79	0.54-1.12
III	1.15	0.85-1.44
IV	1.95	1.45−2.85
Sarcopenia	1.84	1.36-2.78
Anastomotic leak	1.46	1.15-2.94
Pneumonia	2.26	1.41-3.05
Neoadjuvant CR therapy	0.76	0.46-0.97
Immunotherapy	0.89	0.45-1.57

^a^ pTNM and ypTNM

*HR* hazard ratios; *CI* confidence interval

## Discussion

This study shows that sarcopenia is not rare in patients with Siewert type I-II esophageal adenocarcinoma. These subjects have a greater risk of AL, pneumonia, and 90-day mortality, and show lower DFS and OS rates compared to those without sarcopenia. Sarcopenia was found to be an independent predictor of poor OS.

The reported prevalence of sarcopenia is variable depending on patient age, definitions, diagnostic techniques, classifications, and established cutoff points.^18^ Research has highlighted notable differences in sarcopenia prevalence across diverse age cohorts and geographical regions. Specifically, sarcopenia might affect 5–13% of individuals aged 60 to 70 years and up to 50% among of those older than 80 years.^19^ Furthermore, the prevalence of sarcopenia might also be influenced by variations in diagnostic approaches and criteria.^20^ Despite inconsistencies in diagnostic criteria and methodologies used, sarcopenia is commonly identified in patients with esophageal cancer. Haiducu et al. documented a 43.7% prevalence among individuals with gastrointestinal tumors, with esophageal cancer exhibiting the highest rate at 70.4%, largely attributed to the frequent occurrence of dysphagia.^24^ Tan et al. utilized CT data for a retrospective diagnosis of sarcopenia in patients with esophageal cancer, revealing a 75.9% prevalence.^21^ Conversely, a prospective study by Yoshida et al., involving 71 patients and employing bioelectrical impedance analysis (BIA), found a 40.8% prevalence.^22^ A 2023 meta-analysis by Jogiat et al., encompassing 21 studies and 3,966 patients, found that 48.1% of patients were diagnosed with sarcopenia.^23^ In our study, which included only Siewert type I-II esophageal adenocarcinoma, preoperative sarcopenia determined by preoperative CT scan images was diagnosed in 39.7% of patients. In the systematic review and meta-analysis by Park et al, which included both adenocarcinoma and squamous-cell cancer, preoperative sarcopenia was found in 59.7% of patients However, only a subset of the studies assessed sarcopenia with skeletal muscle index.^25^

The specific influence of sarcopenia on short- and long-term postoperative outcomes following esophagectomy is not fully understood. Reduced skeletal muscle mass is associated with impaired respiratory function, including decreased forced expiratory volume in one second and forced vital capacity in older patients.^26^ This may affect the cough reflex, potentially increasing the risk of mucus plugging and atelectasis in the immediate postoperative period. In addition, decreased skeletal muscle function can impact oropharyngeal swallowing (sarcopenic dysphagia), thereby increasing the risk of aspiration.^18^ Sarcopenia has also been linked to changes in immune response, with studies showing a relationship between skeletal muscle mass and immune-modulating cytokines, such as interleukin (IL)-7 and IL-15, which support the development of lymphoblasts and oncological surveillance. Furthermore, sarcopenia and compromised nutritional status are related to lower leukocyte counts, reduced neutrophil margination, and an overall decreased immune response to infectious challenges.^27,28^ These factors may account for the increased rates of pneumonia, sepsis, overall complications, and 90-day mortality observed among our patients with sarcopenia. This is similar to what is described by Elliot et al., concluding that a higher incidence of major postoperative complications (24.5% vs. 11.8%) and pulmonary complications (55.1% vs. 35.7%) in sarcopenic patients.^29^ Similarly, Fehrenbach et al. reported a greater incidence of major complications (HR 2.59) and prolonged hospital stays (32 vs. 19 days) among sarcopenic patients, along with higher pneumonia rates (HR 6.36).^30^ Our study showed that postoperative AL rates were significantly higher in sarcopenic patients (20.8% vs. 11.3%; *p* = 0.02) with sarcopenia being an independent predictor of postoperative AL on multivariate analysis. Differently, Park and colleagues concluded no significant effect of preoperative sarcopenia on postoperative AL (10.9% vs. 10.2%).^25^ Although more research is needed onto this topic, impaired protein synthesis in these patients may determine an increased risk of postoperative anastomotic failure.

In our study, sarcopenia was found to be significantly associated with reduced OS and DFS. Furthermore, sarcopenia was defined as independent predictor or poor OS in the logistic regression analysis (HR = 1.84). These results may be due to sarcopenia reducing the body’s physiological reserve, which is associated with a decreased ability to manage the effects of cancer and its treatments.^20^ Similarly, Park and colleagues concluded that sarcopenia was an independent predictor of poor OS in the regression analysis (HR = 1.68). Also, Tamandl et al. reported inferior survival outcomes in 200 patients who underwent esophageal resection, with a median OS of 31.5 months for sarcopenic patients compared to 76.5 months for nonsarcopenic individuals, identifying sarcopenia as a negative risk factor for survival (HR 1.87).^31^ Similarly, Paireder et al. observed reduced survival in sarcopenic patients compared to their nonsarcopenic counterparts undergoing esophagectomy (median OS, 20.5 months vs. 52.1 months).^32^ Kudou et al. also reported lower 5-year OS rates in patients with sarcopenia following esophagectomy (54.8% vs. 85.5%).^33^ Moreover, a meta-analysis of 41 studies confirmed a significant association between sarcopenia and OS (HR, 1.68).^11^ Lastly, Watanabe et al. performed a retrospective study on 187 patients and found that preoperative sarcopenia assessed by BIA was an independent risk factor for noncancer-specific mortality.^34^ Conversely, other retrospective analyses have failed to establish sarcopenia as a predictor of survival. Elliot et al.^29^ reported no difference in 5-year survival among 252 patients, and Siegal et al.^35^ found no significant impact in their cohort of 173 patients, even after conducting subgroup analyses in the elderly population. Importantly, caution is warranted while interpreting our results, because other significant issues might influence survival in patients with esophageal adenocarcinoma, such as the occurrence of AL, pulmonary complications, centralization in high-volume centers, and appropriate multimodal treatments.^36–41^

Identifying patients with sarcopenia at higher risk for postoperative complications facilitates preoperative interventions to mitigate the detrimental effects of sarcopenia. A comprehensive assessment of these individuals is critical to improve diagnostic criteria and the therapeutic pathway.^18,42^ Prehabilitation is an emerging approach that focuses on enhancing patient’s physical fitness and nutritional reserve in preparation for the physiological stress associated with major surgery.^43^ This approach typically incorporates nutritional support, exercise regimens, and psychological interventions. Intensive perioperative nutritional therapy through enteral or parenteral nutrition, carbohydrate-rich supplements consumed on the morning of surgery, and early mobilization and re-establishment of enteral nutrition within 24 hours after surgery—has been shown to reduce severe complications after esophagectomy, shorten intensive care unit stays, and promote preoperative weight gain.^44,45^ A recent study investigated the effects of prehabilitation on the body composition of esophageal cancer patients. Those who engaged in a comprehensive, personalized exercise program combining aerobic and strength training, retained a higher skeletal muscle index compared with the control group. Furthermore, participants who completed the program showed a significant reduction in visceral adipose tissue.^46^ Consequently, we recommend that sarcopenic patients planning to undergo curative treatment for esophageal cancer participate in prehabilitation program, such as dietitian-led nutritional support and customized exercise programs, during the pretreatment phase. Importantly, the methodology to define sarcopenia should be standardized to reduce statistical heterogeneity and to provide objective information that may help to tailor the most appropriate preoperative management. Longitudinal evaluation of frailty should also be considered in this patient population with the aim to further reduce postoperative mortality and improve survival.^5^

The strengths of this study include its large sample size, the comprehensive collection of clinical, radiologic, and histopathologic data, the focus on patients with esophageal adenocarcinoma, the homogeneity of surgical technique, and the fact that surgery was carried out in two specialized esophageal surgery facilities in tertiary referral hospitals. However, the observational study design can introduce causality and association bias. Additionally, the data collection spanned over a decade during which different regimens of neoadjuvant therapy (CROSS vs. FLOT) or immunotherapy (nivolumab vs. pembrolizumab) were adopted.^39,40^ Therefore, the results of the present study should be interpreted with caution. Due to the retrospective nature of our study, we were unable to fully retrieve data regarding nutrition, nutrition modality (parenteral versus enteral), daily caloric intake, immunonutrition, and other prehabilitation strategies. Furthermore, the findings are reflective of outcomes within a Western population and may not be broadly generalizable. Other clinical examinations, such as skeletal muscle index, handgrip strength, chair stand test, SARC-F questionnaire, CT measurements, or BIA, may be used to quantify sarcopenia more accurately and to identify frailty.

## Conclusions

This study indicates that sarcopenia is a highly prevalent and significant preoperative comorbidity among patients with esophageal adenocarcinoma. Patients with sarcopenia had lower DFS and OS rates compared with those without sarcopenia. Furthermore, sarcopenia was independently associated with postoperative anastomotic leak, pneumonia, 90-day mortality, and poor OS. Evaluating sarcopenia can serve as a complementary tool to current nutritional assessment and prognostic methods, potentially enabling risk factor modification and the early identification of complications.

## Data Availability

The data collected and analyzed during the current review are available from the corresponding author on reasonable request.
